# Cognitive Reserve, Executive Function, and Memory in Parkinson’s Disease

**DOI:** 10.3390/brainsci11080992

**Published:** 2021-07-27

**Authors:** Andrea M. Loftus, Natalie Gasson, Nicole Lopez, Michelle Sellner, Carly Reid, Naomi Cocks, Blake J. Lawrence

**Affiliations:** 1 Discipline of Psychology, School of Population Health, Curtin University, Perth 6148, Australia; n.gasson@curtin.edu.au (N.G.); nicole.lopez@student.curtin.edu.au (N.L.); michelle.sellner@student.curtin.edu.au (M.S.); carly.reid@curtin.edu.au (C.R.); blake.lawrence@curtin.edu.au (B.J.L.); 2 School of Allied Health, Curtin University, Perth 6148, Australia; naomi.cocks@curtin.edu.au

**Keywords:** Parkinson’s disease, cognition, cognitive reserve, executive function, memory

## Abstract

Cognitive impairment is acknowledged as a feature of Parkinson’s disease (PD), and the most common cognitive declines are in executive function (EF) and memory. Cognitive reserve (CR) may offer some protection against cognitive dysfunction in PD. The present study used two proxies of CR (years of education, premorbid IQ) to examine the relationship between CR and (i) EF (ii) memory in a large PD sample (*n* = 334). Two aspects of EF were examined, including verbal fluency and planning skills. Two aspects of verbal memory were examined, including immediate recall and delayed recall. For EF, both CR proxies significantly predicted verbal fluency, but only years of education predicted planning skills. Years of education significantly predicted immediate recall, but premorbid IQ did not. Neither CR proxy predicted delayed recall. These findings suggest that CR, in particular years of education, may contribute to EF and memory function in those with PD. A key finding of this study is the varying contribution of CR proxies to different aspects of the same cognitive domain. The findings indicate that using only one proxy has the potential to be misleading and suggest that when testing the relationship between CR and cognition, studies should include tasks that measure different aspects of the cognitive domain(s) of interest.

## 1. Introduction

Cognitive impairment is now acknowledged as a feature of Parkinson’s disease (PD), although the pattern, rate, and predictors of cognitive decline remain unclear [1,2,3]. As well as making everyday tasks challenging for people with PD and their families, cognitive impairment negatively impacts quality of life and functional independence [4,5]. Given its considerable impact, it is important to understand the factors that may contribute to, or protect against, cognitive impairment in PD. Cognitive reserve (CR) may protect against cognitive impairment associated with a range of different pathologies, including Alzheimer’s disease [6], multiple sclerosis [7,8], and PD [9,10].

The concept of CR stems from the observation that the exact same brain damage can result in individual differences in symptom severity. Whereas ‘brain reserve’ suggests that greater numbers of neurons and synapses (i.e., larger brains) affords protection, CR is an active compensatory process whereby preexisting cognitive resources are used to cope with brain damage [9,11]. CR encompasses a number of different factors, including genetics, environment, education, occupational demands, lifetime experiences, and mental stimulation [12,13,14,15,16]. As it is thought to be multifactorial, cumulative, and experience-dependent, there is no single measure of CR. Indirect proxies are commonly used to measure CR, such as education, intelligence quotient [IQ], occupational attainment, leisure activity, social activity, and physical activity [11,14,15,17,18,19,20,21,22].

Higher CR is associated with lower overall cognitive impairment [6,7,9], but it is unclear which specific aspects of cognition may be protected by CR in PD. The aspects of cognition most impacted by PD are memory and executive function (EF; [1,2,23,24,25,26]). The findings relating to CR and EF are sparse and mixed [9,10,27,28]. Hindle et al. [9] examined the relationship between CR and cognition in PD using educational achievement, socio-occupational status, and current social engagement as early-, mid-, and late-life CR proxies, respectively [9]. A large PD participant sample (*n* = 330) completed the Mini-Mental State Exam (MMSE) and the Addenbrooke’s Cognitive Examination–Revised test. Education and social status were related to baseline overall cognition and EF, but they found no other significant relationships between CR proxies and global cognition over time. Koerts et al. [29] examined the relationship between CR and cognition using level of education and premorbid IQ as CR proxies. They reported that premorbid IQ predicted EF in PD, but not level of education. As a composite score of EF across four different tasks was used, the impact of premorbid IQ on different aspects of EF (e.g., planning, verbal fluency) could not be determined. Rouillard et al. [27] examined the relationship between CR and cognition in PD using four proxies—education, professional experience, and participation in physical and leisure activities across the lifetime. Education was the only significant predictor of EF and its effect was evidenced in only two of the five EF tasks used (updating of working memory, phonemic fluency). This led the authors to suggest that CR might vary both between and within cognitive domains in PD [27]. Ciccarelli et al. [28] used the Cognitive Reserve Index questionnaire and the Brief Intelligence Test to calculate a composite CR score. They reported that higher CR scores were associated with better verbal EF (word fluency, digit span backward), but this was not the case for nonverbal EF (Rey–Osterrieth figure copy, double barrage). The authors attributed this difference to the sensitivity of the former tasks to the fronto-striatal damage associated with PD, particularly in relation to task-switching.

Whereas research examining the relationship between CR and EF or overall cognition in PD exists, studies evaluating CR and memory in PD are sparse. There is evidence in other health conditions that the two are related. CR may buffer memory changes associated with normal healthy ageing [23] and traumatic brain injury [30]. Among those CR studies in PD that have included memory-specific tasks, the findings are mixed. Koerts et al. [29] reported that premorbid IQ predicted semantic memory in PD. However, as a composite score of memory was used, the impact of premorbid IQ on specific aspects of memory could not be determined. Rouillard et al. [23] reported that, out of four CR proxies included in the study, only education predicted episodic memory performance. Ciccarelli et al. [28] reported that CR (composite of two CR proxies) did not predict memory in those with PD.

While there is some research of CR and specific aspects of cognition in PD, this is a developing field. There is still work to be done to improve our understanding of the potential protective role of CR in PD. The present study used two proxies of CR (years of education and premorbid IQ) to examine the relationship between CR and (i) EF (verbal and planning) and (ii) verbal memory (immediate recall, delayed recall) in a large community-based PD population (*n* = 334).

## 2. Materials and Methods

### 2.1. Participants

Participants were recruited as part of an ongoing study by ParkC in Western Australia examining a range of motor and cognitive symptoms of PD. This study was approved by Curtin University Ethics Committee and all research was conducted in accordance with the Declaration of Helsinki. All participants provided written, informed consent. Participants were included in the study only if they had a formal diagnosis of idiopathic PD by a neurologist or geriatrician. Participants were excluded from the study if they self-reported any condition that could interfere with cognitive assessment. Three-hundred and thirty-five participants with idiopathic PD were included in the study. In accordance with Emre et al. [24], no participants had PD dementia at study entry. The mean Mini Mental State Examination [25] score at study entry was 26.99 (SD = 2.54). Participants were people with PD, recruited through convenience sampling from Parkinson’s Western Australia and through advertising in community newsletters, newspapers, social media sites (facebook, twitter), and local radio.

### 2.2. General Procedure

Participants were mailed a questionnaire pack before undertaking a neuropsychological assessment. All participants were tested in the “on” state—approximately 1 h post-PD-medications. Assessments took approximately 2.5 h, during which the participant completed a range of measures of neuropsychological measures, including those described below.

### 2.3. Measures

Age, sex, disease duration, and levodopa equivalent dose (LED) [26] were collected via self-report questionnaire and were used as demographic characteristics. Years of education was collected via self-report questionnaire at the same time as the aforementioned demographic measures, and was used a proxy of CR.

The Australian National Adult Reading Test (AUSNART) [31] was used as a proxy for CR. The AUSNART is used to estimate premorbid verbal IQ and requires participants to read 64 non-phonetic words aloud. An error score was used to derive a verbal IQ estimate using a regression equation [32]. The AUSNART was used as the ability to read non-phonetic words often remains intact despite brain pathologies, and the AUSNART has strong internal consistency (.94) and sound-convergent validity with other established IQ measures (0.61 to 0.76) [32].

The Stockings of Cambridge Task (CANTAB™) was used to measure nonverbal EF, specifically planning skills. Three colored balls are presented at the top of the screen in a specific (target) configuration. Three identical balls are simultaneously presented at the bottom of the screen, in a different configuration. Participants are required to match the configuration of the balls at the bottom of the screen with the configuration presented at the top of the screen. Participants are instructed that they have a limited number of moves in which to match the two sets. Task complexity is determined by the minimum number of moves required to solve the task, which ranges from one to five. The proportion of problems solved in the minimum number of moves was calculated across 12 trials (two two-move trials, two three-move, four four-move, and four five-move). Higher scores indicate greater planning ability. This test has been used extensively to measure EF in PD.

The Controlled Oral Word Association Task [33] was used to measure verbal EF. Participants are asked to generate as many words as possible beginning with the letters F, A, and S, each in 60 s. Verbal EF was calculated as the total number of correct responses across all three trials, with higher scores indicating greater verbal EF.

The Hopkins Verbal Learning Test—Revised [34] was used to measure both immediate and delayed recall for verbal memory. Twelve nouns (four words from three semantic categories) are read to the participant three times. The same list (mammals, precious gems, residences) was used for all participants. Immediately after each reading, the participant is asked to recall as many words as possible. The total correct responses over trials 1–3 was calculated, with higher scores indicative of greater immediate verbal memory recall. As a measure of delayed recall, the participant is asked to recall as many words on the list 25 min after the last immediate recall trial (trial 3). The total correct responses for this trial (trial 4) were calculated, with higher scores indicative of greater delayed recall.

### 2.4. Statistical Analysis

Hierarchical regressions were used to determine whether proxies for CR were associated with cognition in PD. Four hierarchical regressions were conducted in total, one for each of the four cognitive measures (SOC, COWAT, HVLT-R immediate, HVLT-R delayed). Only the demographic characteristics (i.e., sex, age, disease duration, and levodopa equivalent dose) significantly correlated with the outcome variable were included as covariates in each model. For each regression model, covariates were entered at step one and CR proxies were entered as criterion predictors at step two.

## 3. Results

Demographic characteristics, test scores, and CR are presented in Table 1. Table 2 provides Pearson *r* correlations between demographic characteristics and outcome variables.

Four separate hierarchical regressions examined whether proxies for CR were associated with cognition in PD (see Table 3). The first regression model included SOC as the outcome variable, age as a control variable, and years of education and premorbid IQ as predictors. For SOC, years of education accounted for a statistically significant 2.2% of the variance in scores on the SOC task, and premorbid IQ did not account for any variance. The second regression model included COWAT as the outcome variable, age and sex as control variables, and years of education and premorbid IQ as predictors. For COWAT, years of education and premorbid IQ accounted for a statistically significant 2% and 1.6% of the variance, respectively. The third regression model included HVLT-R immediate as the outcome variable, age and sex as control variables, and years of education and premorbid IQ as predictors. For HVLT-R immediate, years of education accounted for a statistically significant 2.5% of the variance in scores. The final regression model included HVLT-R delayed as the outcome variable, age and sex as control variables, and years of education and premorbid IQ as predictors. For HVLT-R delayed, neither CR proxy significantly predicted variance in scores.

## 4. Discussion

The present study examined the relationship between CR and cognition in PD, using years of education and premorbid IQ as CR proxies. Of particular interest was the relationship between CR and two different cognitive domains—EF and memory. Two aspects of EF were examined, including verbal fluency (using the COWAT) and planning skills (using the SOC). Both CR proxies significantly predicted verbal fluency, but only years of education predicted planning skills. Two aspects of memory were examined, including immediate recall and delayed recall (using the HVLT-R). Years of education significantly predicted immediate recall, but premorbid IQ did not. Neither CR proxy predicted delayed recall. Increased CR (i.e., higher years of education and higher premorbid-IQ) was associated with increased performance for all cognitive tests. These findings are consistent with previous PD research and suggest that CR, in particular years of education, may contribute to EF and memory function in those with PD.

The finding that years of education, but not premorbid IQ (as measured by the AUSNART), predicted planning skills is worthy of consideration. The SOC is a task that requires the participant to choose the necessary actions to reach a goal, decide the right order, assign each task to the proper cognitive resources, and establish and execute a plan of action [35]. There is no verbal component to the SOC. Conversely, as a measure of verbal fluency, the COWAT is a task that requires the participant to retrieve information from their mental lexicon, within restricted parameters, and produce a verbal response [33]. Verbal fluency tasks can be semantic (words from a particular category) or phonemic (words that begin with particular sounds). In this study, a phonemic fluency task was used. Both CR proxies significantly predicted verbal fluency. When we consider the fundamental disparities between these tasks, it makes sense that the two aspects (and therefore measures) of CR might differentially predict EF task performance. Verbal (letter) fluency was the only task predicted by premorbid IQ in the present study. The premorbid IQ test used in the current study was a non-phonetic reading task. To succeed in this task, participants must have previously been exposed to both the verbal and orthographic form of that word, have stored the verbal and orthographic form of that word, and be able to access and retrieve that word during the task. Task performance is therefore highly dependent on one’s vocabulary. Letter fluency is also dependent on vocabulary size in adults [36,37]. The task requires a participant to suppress the words that are semantically associated, and focus on the retrieval of the phonemic form of the words. Those with a larger vocabulary will therefore have to engage in more suppression, as more words will be activated. While working memory does play a role in letter fluency tasks, as the task requires participants to remember the words they have already said, vocabulary size is a more important predictor of task performance. It has been reported that having a greater vocabulary offsets the effects of cognitive decline associated with aging on letter fluency tasks in healthy adults [36]. While the HLVT-R is also a verbal task, unlike the letter fluency task it is not dependent on vocabulary size. The HVLT-R is more dependent on working memory and recall, hence premorbid IQ did not predict scores on the HVLT-R in the present study. In light of this, a nonverbal assessment of premorbid IQ could be considered for inclusion in future research. This poses a challenge, as verbal tests are typically easier to use in a population with issues relating to movement and/or comprehension, since they do not require a motor response and/or reading instructions.

Neither CR proxy predicted delayed recall on the HVLT-R. Delayed recall is frequently used as a screening measure for dementia [38,39] and is included in global measures of cognition, such as the MMSE. Delayed recall relies more heavily on verbal memory retrieval than immediate recall, and requires that the participant learns and consolidates (to long term memory) a verbal list of words. In comparison, immediate recall involves more working verbal memory. There is no clear explanation as to why CR (specifically, years of education) might contribute more to one aspect of verbal memory than another, and this is something that could be examined further in future research by including a range of different memory tasks.

A key finding of this study is the varying contribution of CR proxies to different aspects of the same cognitive domain. Among studies examining CR and cognition, many used only one proxy [9,10]. The current findings indicate that using only one proxy has the potential to be misleading, as years of education and premorbid IQ in the present study contributed differently to specific aspects of cognition. Years of education predicted more variance in EF and memory than premorbid IQ. This is not to say that years of education is a more valid CR proxy than premorbid IQ, but it does question the value of combining CR proxies into one representative measure. Combining years of education and premorbid IQ in the present study may have changed the findings, and information about the distinct contribution of CR proxies to different aspects of cognition may have been lost.

The present findings also suggest that when testing the relationship between CR and cognition, studies should consider including measures of specific aspects of cognition—especially those impacted in the population they are exploring. The aspects of cognition most impacted by PD are memory and executive function [1,2,40,41,42] and these aspects of cognition are arguably more complex than one representative task. While global measures of cognition are reliable indicators of overall cognitive decline, they are not used to identify specific cognitive impairment in PD [43]. For example, when identifying mild cognitive impairment in PD (PD-MCI), a Movement Disorder Society (MDS) taskforce requires at least two tests for each of the five cognitive domains of executive function, attention and working memory, language, memory, and visuospatial function [43]. It is not advisable to use a measure of global cognition, as the impaired cognitive domain(s) cannot be identified. Ideally, the present study would have included set-shifting and inhibition tasks (aspects of EF), and a nonverbal memory task. However, this was beyond the scope of this study and poses a problem of increased participant burden.

The present study is unique in that it included two proxies of CR, two tests per cognitive domain, control variables associated with cognitive function in PD, and a large sample size. There are, however, a number of limitations to this study that must be considered. First, as this was part of a longitudinal study of Parkinson’s and this manuscript is a retrospective exploration of the data collected at study entry, we were limited in the CR proxies available to us. Ideally, we would have also included a specific measure of CR such as the Cognitive Reserve Index Questionnaire [44]. We were limited to two proxies (years of education, premorbid IQ), so were unable to explore other measures of CR in Parkinson’s. The participants in this study were active in the community and in managing their disease, with a majority volunteering from Parkinson’s WA. This might have impacted the results, as social and physical engagement are amongst the CR proxies not considered in the present study. This speaks to the challenge of conceptualizing and measuring CR, as there are a range of different CR proxies that could be at play and these should all ideally be measured and included in analyses. Furthermore, the distribution of scores on the MMSE suggests that a small proportion of participants may have been classified as PD-MCI if they had been assessed with a more extensive battery of neuropsychological tests, which is important to consider in light of these preliminary results. While the present study goes some way to expanding our understanding of CR and cognition in PD, more research is needed. Only with a clearer understanding of the role of CR can we develop appropriate and effective enrichment programs for those at risk of cognitive impairment.

## Figures and Tables

**Table 1:** Demographic characteristics and cognition scores, stratified by sex.

	Male (*n* = 221)		Female (*n* = 114)			Total (*n* = 335)	
	*M*	*SD*	*M*	*SD*	*p ^+^*	*M*	*SD*
Age (years)	67.25	9.56	64.64	9.25	0.017 *	66.37	9.49
Disease duration (years)	5.03	4.72	6.32	5.21	0.023 *	5.46	4.91
Levodopa Equivalent Dose	574.79	420.96	521.61	484.81	0.30	556.70	442.39
Education (years)	12.97	3.69	13.07	3.28	0.80	13	3.54
Premorbid IQ	107.04	10.86	106.37	7.42	0.55	106.81	9.79
MMSE	26.66	2.614	27.61	2.31	0.001 *	26.99	2.54
SOC	6.88	2.43	7.12	2.30	0.38	6.96	2.38
COWAT	35.83	13.19	41.70	12.44	0.001 **	37.83	13.18
HVLT-R (immediate)	21.33	6.81	26.00	5.70	0.001 **	22.92	6.80
HVLT-R (delayed)	7.46	2.99	9.19	2.58	0.001 **	8.05	2.96

^+^ = statistical significance from an independent samples t-test; * = *p* < 0.05; ** = *p* < 0.001; *M* = mean; *SD* = standard deviation; MMSE = Mini-Mental State Exam; SOC = Stockings of Cambridge; COWAT = Controlled Oral Word Association Test; HVLT-R = Hopkins Verbal Learning Test—Revised.

**Table 2:** Correlation matrix (r) of demographic characteristics and outcome variables.

*n* = 335	1	2	3	4	5	6	7	8	9	10	11
1. age (years)	-										
2. sex	−0.10	-									
3. disease duration	−0.05	0.12	-								
4. LED	−0.08	−0.07	0.42 **	-							
5. education (years)	−0.20 **	0.01	−0.01	−0.01	-						
6. premorbid IQ	−0.10	−0.04	−0.04	−0.04	0.65 **	-					
7. MMSE	−0.26 **	0.18 **	−0.04	−0.04	0.28 **	0.31 **	-				
8. SOC	−0.36 **	0.03	0.01	0.03	0.21 **	0.08	0.26 **	-			
9. COWAT	−0.17 **	0.22 **	0.00	−0.03	0.34 **	0.30 **	0.25 **	0.21 **	-		
10. HVLT-R (imm)	−0.36 **	0.32 **	−0.12 *	−0.05	0.29 **	0.13 *	0.40 **	0.28 **	0.43 **	-	
11. HVLT-R (del)	−0.30 **	0.27 **	−0.06	−0.01	0.26 **	0.17 *	0.44 **	0.31 **	0.35 **	0.77 **	-

* = *p* < 0.05; ** = *p* < 0.001; LED = Levodopa Equivalent Dose; MMSE = Mini Mental State Examination; SOC = Stockings of Cambridge; COWAT = Controlled Oral Word Association Test; HVLT-R (imm)/(del) = Hopkins Verbal Learning Test—Revised, immediate/delayed.

**Table 3:** Unstandardized (B) and standardized (β) regression coefficients and squared semi-partial correlations (sr2) for cognitive reserve proxies predicting cognition.

Outcome	Step	Predictor	*B*	95% CI		β	*t*	*p*	*sr* ^2^
SOC	1	age	−0.11	−0.13	−0.08	−0.43	−8.73	0.001 **	0.18
	2	age	−0.10	−0.12	−0.08	−0.40	−8.07	0.001 **	0.15
		years of education	0.13	0.05	0.22	0.20	3.082	0.002 *	0.02
		premorbid IQ	−0.02	−0.05	0.02	−0.06	−0.96	0.34	0.00
COWAT	1	age	−0.35	−0.49	−0.21	−0.25	−4.81	0.001 **	0.06
		sex	4.95	2.09	7.82	0.18	3.40	0.001 *	0.03
	2	age	5.24	−0.41	−0.13	−0.19	−3.86	0.001 **	0.04
		sex	0.69	2.53	7.95	0.19	3.80	0.001 **	0.04
		years of education	0.23	0.22	1.17	0.19	2.88	0.004 *	0.02
		premorbid IQ	5.24	0.06	0.40	0.17	2.64	0.009 *	0.01
HVLT-R (immediate)	1	age	−0.30	−0.36	−0.23	−0.41	−8.72	0.001 **	0.17
		sex	3.90	2.57	5.24	0.27	5.76	0.001 **	0.07
	2	age	−0.27	−0.33	−0.20	−0.37	−7.93	0.001 **	0.13
		sex	3.94	2.64	5.24	0.27	5.96	0.001 **	0.07
		years of education	0.40	0.17	0.63	0.21	3.48	0.001 *	0.02
		premorbid IQ	0.00	−0.08	0.08	0.00	0.02	0.98	0.00
HVLT-R (delayed)	1	age	−0.11	−0.14	−0.08	−0.34	−6.66	0.001 **	0.12
		sex	1.46	0.83	2.10	0.23	4.53	0.001 **	0.05
	2	age	−0.10	−0.13	−0.06	−0.31	−5.95	0.001 **	0.09
		sex	1.50	0.87	2.12	0.24	4.71	0.001 **	0.06
		years of education	0.10	−0.01	0.21	0.12	1.84	0.07	0.01
		premorbid IQ	0.02	−0.02	0.06	0.08	1.15	0.25	0.00

CI = confidence interval; SOC = Stockings of Cambridge; COWAT = Controlled Oral Word Association Test; HVLT-R = Hopkins Verbal Learning Test—Revised; * = *p* < 0.05; ** = *p* < 0.001.

## Data Availability

This data is not publicly available due to ethical reasons and the longitudinal nature of the study.
